# Albany Co. Medical Society

**Published:** 1872-12

**Authors:** 


Albany County Medical Society.—The report of the meeting of this Society for December, reached us too late for publication in this Journal, but will appear in the January number, together with the report of the meetings for that month.

This Society is fully awake to its work, and its meetings always call forth many valuable and scientific papers. The able and efficient Secretary, Dr. F. C. Curtis, of Albany, has undertaken to furnish our readers with reports of the monthly meetings, and we congratulate our readers upon the opportunity afforded them of reading the valuable papers and discussions presented to this society. We are personally under many obligations to Dr. Curtis for his kindness.

